# Genomic Identification and Expression Analysis of the Phosphate Transporter Gene Family in Poplar

**DOI:** 10.3389/fpls.2016.01398

**Published:** 2016-09-16

**Authors:** Chunxia Zhang, Sen Meng, Mingjun Li, Zhong Zhao

**Affiliations:** ^1^College of Forestry, Northwest A&F UniversityYangling, China; ^2^State Key Laboratory of Soil Erosion and Dryland Farming on the Loess Plateau, Northwest A&F UniversityYangling, China; ^3^College of Horticulture, Northwest A&F UniversityYangling, China

**Keywords:** phosphate transporter, poplar, genome-wide analysis, expression profile, phosphate levels, drought

## Abstract

Inorganic phosphate is one of key macronutrients essential for plant growth. The acquisition and distribution of phosphate are mediated by phosphate transporters functioning in various physiological and biochemical processes. In the present study, we comprehensively evaluated the phosphate transporter (PHT) gene family in the latest release of the *Populus trichocarpa* genome (version 3.0; Phytozome 11.0) and a total of 42 *PHT* genes were identified which formed five clusters: *PHT1, PHT2, PHT3, PHT4*, and *PHO*. Among the 42 *PHT* genes, 41 were localized to 15 *Populus* chromosomes. Analysis of these genes led to identification of 5–14 transmembrane segments, most of which were conserved within the same cluster. We identified 234 putative *cis* elements in the 2-kb upstream regions of the 42 *PHT* genes, many of which are related to development, stress, or hormone. Tissue-specific expression analysis of the 42 *PtPHT* genes revealed that 25 were highly expressed in the roots of *P. tremula*, suggesting that most of them might be involved in Pi uptake. Some *PtPHT* genes were highly expressed in more than six of the twelve investigated tissues of *P. tremula*, while the expression of a few of them was very low in all investigated tissues. In addition, the expression of the *PtPHT* genes was verified by quantitative real-time PCR in four tissues of *P. simonii.* Transcripts of *7 PtPHT* genes were detected in all four tested tissues of *P. simonii*. Most *PtPHT* genes were expressed in the roots of *P. simonii* at high levels. Further, *PtPHT1.2* and *PtPHO9* expression was increased under drought conditions, irrespective of the phosphate levels. In particular, *PtPHT1.2* expression was significantly induced by approximately 90-fold. However, the transcriptional changes of some *PtPHT* genes under drought stress were highly dependent on the phosphate levels. These results will aid in elucidation of the functions of *PtPHT* in the growth, development, and stress response of the poplar plant.

## Introduction

Phosphorus is a major macronutrient required for plant growth and development. It constitutes up to 0.2% of the dry weight of plant cells and consequently is required in significant quantities (Schachtman et al., 1998). However, it is often a limiting factor for plant growth because the soil inorganic phosphate (Pi) concentration is usually lower than 10 μM (Shen et al., 2011). Plants have adopted several strategies to increase the acquisition of poorly available P source (Bucher, 2007). For example, plants could produce root exudates such as organic acids, sugars, and phenolic acids in the rhizosphere to improve the acquisition of poorly available P from soil (Cesco et al., 2010; Valentinuzzi et al., 2015). Additionally, plants have evolved specific transport systems for the uptake and partitioning of Pi (Poirier and Bucher, 2002). Both low- and high-affinity Pi-transporter systems have been identified in plants (Clark et al., 2000; Misson et al., 2004). The genes encoding for low-affinity Pi transport are constitutively expressed and function at relatively high Pi in the millimolar range, whereas high-affinity Pi transporters are transcriptionally induced at low Pi levels in the micromolar range (Raghothama, 2000).

Generally, the phosphate transporters are classified into PHT1, PHT2, PHT3, and PHT4 families (Rausch and Bucher, 2002; Liu et al., 2011). PHT1s belonging to the major facilitator superfamily are homologs of the yeast PHO84 Pi transporter (Pao et al., 1998). Since the initial identification of PHT1 genes in *Arabidopsis* (Muchhal et al., 1996), homologs of PHT1 transporters have been characterized in many species, such as tomato (Chen et al., 2014), rice (Liu et al., 2011), and poplar (Loth-Pereda et al., 2011). The PHT1 family, as H^+^/Pi symporters, has been reported to include proteins involved in the Pi uptake from the soil and distribution within plants (Nussaume et al., 2011). Regarding the PHT2 family of Pi transporters, one member (PHT2;1) has been functionally studied in *Arabidopsis* (Daram et al., 1999). PHT2 family proteins function as H^+^/Pi cotransporters in the plastids of plants (Versaw and Harrison, 2002). In addition, the PHT3 family was reported after the cloning of the first putative mitochondrial Pi transporter gene in plants (Takabatake et al., 1999). The PHT3 family, which are supposed to act as a Pi/H^+^ symporter Pi/OH^−^ antiport, catalyze Pi/Pi exchange between the matrix and cytosol (Stappen and Krämer, 1994). This family included at least three members in *Arabidopsis* to date, and all of them show high conservation within the mitochondrial transporter family (Rausch and Bucher, 2002). The PHT4 gene family, which includes six members in *Arabidopsis*, has been reported to be involved in the Pi transport in plastids and the Golgi apparatus (Guo et al., 2008). Recently, the PHO1 gene in Arabidopsis was identified by a map-based positional cloning strategy (Hamburger et al., 2002). The members of PHO1 family were mainly expressed in root stellar cells and in the lower part of the hypocotyl, which is consistent with the role of PHO1 in the loading of Pi to the xylem (Poirier and Bucher, 2002). The expression patterns of 11 PHO1 homologs in the *Arabidopsis* genome suggest that PHO1 proteins likely play a role in transferring Pi to the vascular cylinders in various tissues, as well as in acquiring Pi into cells, such as pollen, root epidermal cells, and cortical cells (Wang et al., 2004). Most studies of PHT have focused on a single cluster of the PHT family, such as the PHT1 family (Loth-Pereda et al., 2011; Nussaume et al., 2011); however, to our knowledge, there is no report of the whole PHT family, including PHT1, PHT2, PHT3, PHT4, and PHO, in any plant species. In addition, there are few reports of coordinated expression of the entire set of PHTs on a global scale in woody tree species.

Poplar is a model woody tree species used in functional genomic studies due to its small genome, easy transformation, fast growth, and vegetative propagation (Brunner et al., 2004a). *Populus simonii* is an important poplar species in the northwestern area of China with a wide distribution, and it is capable of growing under low- or high-Pi conditions (Weisgerber and Han, 2001). In this study, we conducted comprehensive genome-wide analysis to identify phosphate transporter gene family in poplar, including PHT1, PHT2, PHT3, PHT4, and PHO, in the *P. trichocarpa* genome (version 3.0; Phytozome 11.0); in addition, we analyzed the phylogeny, gene structures, genomic locations, and *cis* elements of these genes. We also investigated the expression patterns of all of the *PHT* genes in various tissues, as well as their expression profiles at different Pi levels (Pi starvation, low and high Pi levels) in *P. simonii*. Moreover, we examined the transcriptional profiles of *PHT* genes in *P. simonii* roots under drought conditions. The results of this study may provide a basic foundation for future functional genomic studies of the PHT family in poplar.

## Materials and methods

### Plant seedlings and growth conditions

The cuttings of *P. simonii* (*ca*. 15 cm in length, 2 cm in diameter) were rooted in pots (10 L) filled with fine sand. The plants were cultivated in a glasshouse (natural light, 75% relative humidity) and irrigated with 50 ml nutrient solution (Hoagland and Arnon, 1950) (10 μM EDTA·FeNa, 5 μM MnSO_4_·H_2_O, 1 μM ZnSO_4_·7H_2_O, 1 μM CuSO_4_·5H_2_O, 30 μM H_3_BO_3_, 0.5 μM H_2_MoO_4_, 1000 μM MgSO_4_·7H_2_O, 1000 μM CaCl_2_, 1000 μM Na_2_SO_4_, and 1000 μM NH_4_NO_3_, pH 6.5) containing Pi at one of three different levels (0 μM, 1000 μM, and 3000 μM KH_2_PO_4_) separately every other day. Each Pi treatment was applied to 12 plants for a period of 8 weeks. In addition, a phosphate-free solution was prepared by replacing KH_2_PO_4_ with K_2_SO_4_. Six plants for each Pi treatment were subjected to drought stress for 2 weeks before harvesting, and the other six plants for each treatment were not subjected to drought stress and served as controls. After the treatments, whole plantlets were harvested and separated into roots, stems, new leaves, and old leaves. Then, samples from the same tissues were pooled for each treatment, frozen in liquid nitrogen for further analysis.

### Identification and phylogenetic analysis of *PHT* gene family members in *populus*

To identify phosphate transporters in poplar, the reported protein sequences from both *Arabidopsis* and *Oryza sativa* were used as query sequence with *E*-value cutoff set as 1e^−10^ to perform a local BLASTP against *P. trichocarpa* JGI gene catalog (phytozome v11.0, https://phytozome.jgi.doe.gov/pz/portal.html), respectively. The choice of candidate phosphate transporters was based on the *E*-value, the sequence homology value (>60%) and the value of score (>450). All corresponding DNA and protein sequences of poplar phosphate transporters were obtained from Phytozome 11.0 (http://phytozome.jgi.doe.gov/pz/portal.html) and also verified with the Pfam database (http://pfam.xfam.org/). Various splicing variants of one gene and incomplete genes with short length were discarded. In addition, redundant sequences were removed, and the remaining sequences were used in further analyses.

For each putative protein, the grand average of hydropathicity (GRAVY) was calculated using ProtParam (http://web.expasy.org/protparam/). Further, TMpred (http://www.ch.embnet.org/software/TMPRED_form.html) was used to predict the transmembrane domains (TMDs) of each PHT protein. In addition, the subcellular location of each PHT was predicted using WoLF PSORT (http://www.genscript.com/wolf-psort.html). Multiple amino acid alignments of the PHTs were generated using ClustalW (Larkin et al., 2007). Phylogenetic tree was constructed using the neighbor-joining method with the 1000 bootstrap in MEGA 6 (http://www.megasoftware.net/).

### Chromosomal locations and gene structures

The chromosomal locations and gene structures of the *PHT* genes were obtained from Phytozome 11.0. The chromosomal locations of the *PHT* genes were mapped with MapDraw program (Liu and Meng, 2003). The exon/intron organization of the individual *PHT* gene was drawn using Gene Structure Display Server (GSDS) program (Hu et al., 2015).

### *Cis*-regulatory element identification

Promoter sequences located 2 kb upstream of the transcription start sites (ATG codons) of the *Populus* PHT genes were retrieved from the *Populus trichocarpa* genome v3.0 in Phytozome 11.0 (http://phytozome.jgi.doe.gov/pz/portal.html). The *cis*-regulatory elements were scanned in PLACE (Higo et al., 1999).

### RNA isolation and quantitative real time PCR analysis

Total RNA was isolated from roots, stems, new leaves, and old leaves using a plant RNA extraction kit (R6827, Omega Bio-Tek, GA, USA) according to the manufacturer's instructions. The first-strand cDNA was synthesized using a PrimeScript RT Reagent Kit (DRR037S, Takara, Dalian, China) following the removal of trace genomic DNA using DNase I (E1091, Omega Bio-Tek). The specific primers (Table S1) for quantitative real time PCR analysis were designed by Primer Premier 6.0 (Premier Biosoft, Palo Alto, CA, USA). We performed PCR in a 20 μl reaction mixture containing 10 μl of 2X SYBR Green Premix Ex Taq II (Bioteke, China), 2 μl of cDNA, and 1 μl of 20 mM of each primer (Table S1) using a Roche LightCycle 96 machine (Roche, Germany). PCR amplification was performed under the following conditions: one cycle of 2 min at 95°C, followed by 45 cycles at 95°C for 10 s, 55°C for 20 s, and 72°C for 20 s. Actin2/7 was used as a reference gene (Brunner et al., 2004b). Three biological replicates with three technical replicates were assayed for each sample. Reactions for the reference gene were included in each plate. The relative expression levels of all the *PtPHT* genes were calculated using the 2^−ΔΔCT^ method (Livak and Schmittgen, 2001).

## Results

### Identification and phylogenetic analysis of the *PtPHT* gene family

We identified a total of 42 putative *PtPHT* genes, including 12 *PHO*, 14 *PtPHT1*, 2 *PtPHT2*, 6 *PtPHT3*, and 8 *PtPHT4* genes from the genome of *P. trichocarpa* according to the full *AtPHT* family in *Arabidopsis*f (Table 1). The lengths of the encoded proteins ranged from 314 amino acids to 802 amino acids, and their sequences contained 5 to 14 TMDs. The molecular weight (MW) of 42 putative proteins ranged from 33.5 to 93.5 kDa. The GRAVY value of putative phosphate transporter protein (PHT family) was positive and ranged from 0.128 to 0.795, while that of PHO family was negative ranging from −0.255 to −0.064.

**Table 1:** **Phosphate transporter genes identified in *Populus***.

**Gene name**	**ID**	**Gene family**	**Genome location**	**ORF (bp)**	**Protein size**	**MW (kDa)**	**PI**	**GRAVY**	**Exon Number**	**TMD (Inside to outside)**	**TMD (Outside to inside)**	**Subcellular location prediction**
PtPHT1.1	Potri.002G038900	PHT1	Chr02:2502616-2504187	1572	523	57.3	9	0.425	1	12	12	plas: 8, chlo: 4, cyto: 2
PtPHT1.2	Potri.005G223600	PHT1	Chr05:23449050-23451047	1620	539	59.5	8.91	0.318	1	12	13	plas: 8, E.R.: 2, chlo: 1, cyto: 1,extr: 1
PtPHT1.3	Potri.005G223500	PHT1	Chr05:23439709-23443572	1620	539	59.5	8.79	0.311	1	12	13	plas: 8, golg: 2, cyto: 1, extr: 1,vacu: 1
PtPHT1.4	Potri.001G318500	PHT1	Chr01:32324342-32326669	1608	535	58.5	8.4	0.340	1	12	12	plas: 9, cyto: 2, E.R.: 2
PtPHT1.5	Potri.010G071700	PHT1	Chr10:9852748-9854632	1611	536	58.6	9.22	0.368	1	13	12	plas: 9, golg: 2, cyto: 1, vacu: 1
PtPHT1.6	Potri.010G071600	PHT1	Chr10:9839165-9841828	1611	536	58.5	9.09	0.395	1	13	11	plas: 9, golg: 2, cyto: 1, vacu: 1
PtPHT1.7	Potri.010G072000	PHT1	Chr10:9880373-9882023	1611	536	58.5	9.01	0.385	1	13	11	plas: 10, E.R.: 2, cyto: 1
PtPHT1.8	Potri.010G071500	PHT1	Chr10:9828080-9832093	1311	436	48.4	8.92	0.383	1	10	11	plas: 7, vacu: 3, E.R.: 2, cyto: 1
PtPHT1.9	Potri.005G175500	PHT1	Chr05:19108301-19109905	1605	534	58.3	9.01	0.391	1	12	13	plas: 10, vacu: 2, cyto: 1
PtPHT1.10	Potri.005G175700	PHT1	Chr05:19114828-19116455	1602	533	58.2	9.1	0.392	1	12	13	plas: 11, cyto: 1, vacu: 1
PtPHT1.11	Potri.015G022800	PHT1	Chr15:1767428-1769014	1587	528	58.4	8.91	0.379	1	12	13	plas: 8, vacu: 2, chlo: 1, cyto: 1,E.R.: 1
PtPHT1.12	Potri.019G061900	PHT1	Chr19:9458594-9462206	1596	531	57.5	8.6	0.295	3	12	11	plas: 7, cyto: 4, chlo: 2
PtPHT1.13	Potri.002G005500	PHT1	Chr02:301928-304487	1587	528	59.1	8.53	0.308	2	12	12	plas: 5, cyto: 3, chlo: 2, vacu: 2,E.R.: 1
PtPHT1.14	Potri.005G256100	PHT1	Chr05:25614279-25616906	1587	528	59.2	8.69	0.279	2	12	12	plas: 5, chlo: 3, cyto: 2, vacu: 2,E.R.: 1
PtPHT2.1	Potri.008G186600	PHT2	Chr08:12862580-12864052	963	320	33.5	9.7	0.795	2	9	7	chlo: 11.5, chlo_mito: 6.5, vacu: 2
PtPHT2.2	Potri.010G046300	PHT2	Chr10:7686651-7690278	1773	590	62.6	9.45	0.482	3	14	12	chlo_mito: 6.5, chlo: 6, mito: 5, plas: 2
PtPHT3.1a	Potri.001G322300	PHT3	Chr01:32668358-32672500	1101	366	39.1	9.38	0.208	6	6	6	chlo: 12, nucl: 1
PtPHT3.1b	Potri.017G060800	PHT3	Chr17:5604608-5608548	1125	374	39.7	9.33	0.201	6	6	6	chlo: 10, nucl: 1, mito: 1, extr: 1
PtPHT3.2a	Potri.012G105100	PHT3	Chr12:12918455-12920626	1095	364	39	9.25	0.128	6	6	7	extr: 6, chlo: 4, mito: 2, vacu: 1
PtPHT3.2b	Potri.015G104400	PHT3	Chr15:12253046-12256542	1080	359	38.6	9.22	0.138	6	5	6	chlo: 7, extr: 3, cyto: 2, mito: 2
PtPHT3.3a	Potri.004G207200	PHT3	Chr04:21606560-21611561	945	314	34.3	9.31	0.095	6	5	5	chlo: 4, pero: 3, cyto: 2, plas: 2,nucl: 1, mito: 1
PtPHT3.3b	Potri.005G098800	PHT3	Chr05:7516204-7519482	957	318	35.3	9.33	0.132	6	6	6	cyto: 7, pero: 3, chlo: 2, nucl: 2
PtPHT4.1a	Potri.001G249800	PHT4	Chr01:25927053-25932436	1557	518	57	7.02	0.305	10	10	12	chlo: 5, E.R.: 3, nucl: 2, mito: 2,cyto: 1
PtPHT4.1b	Potri.009G043800	PHT4	Chr09:5006795-5012311	1563	520	57.6	8.51	0.293	10	10	11	plas: 8, E.R.: 4, vacu: 2
PtPHT4.2	Potri.016G111000	PHT4	Chr16:11363273-11368483	1542	513	55.5	9.96	0.428	7	10	12	chlo: 13
PtPHT4.3	Potri.001G248200	PHT4	Chr01:25800713-25804277	1587	528	58	10.07	0.396	8	10	11	chlo: 9, mito: 4
PtPHT4.4	Potri.014G085700	PHT4	Chr14:6748664-6755569	1743	580	63.5	9.36	0.311	11	13	13	plas: 9, E.R.: 3, chlo: 1
PtPHT4.5a	Potri.006G062300	PHT4	Chr06:4567609-4575958	1632	543	59	7.62	0.302	16	10	11	chlo: 6, plas: 5, E.R.: 3
PtPHT4.5b	Potri.018G121600	PHT4	Chr18:14533065-14541167	1662	553	61.1	8.32	0.184	15	9	9	chlo: 6, plas: 4, E.R.: 2, mito: 1
PtPHT4.6	Potri.009G168200	PHT4	Chr09:12785539-12788684	1329	442	48.3	9.76	0.565	1	12	12	plas: 7, chlo: 4, E.R.: 3
PtPHO1	Potri.008G090300	PHO	Chr08:5657579-5663592	2310	769	88.7	9.2	−0.255	13	9	9	plas: 11, E.R.: 1, pero: 1
PtPHO2	Potri.008G090400	PHO	Chr08:5664558-5668256	2388	795	92.7	9.58	−0.153	12	10	10	plas: 11, nucl: 1, E.R.: 1
PtPHO3	Potri.008G110800	PHO	Chr08:7065812-7071152	2406	801	93.5	8.9	−0.257	14	10	9	plas: 7, nucl: 2, vacu: 2, E.R.: 2
PtPHO4	Potri.008G169400	PHO	Chr08:11569013-11576712	2316	771	89.1	9.35	−0.082	15	8	9	plas: 9, nucl: 2, vacu: 2
PtPHO5	Potri.010G069000	PHO	Chr10:9616013-9624357	2322	773	90	9.64	−0.085	15	8	8	plas: 11, vacu: 2
PtPHO6	Potri.010G132300	PHO	Chr10:14708712-14714441	2379	792	92.5	8.88	−0.218	14	8	9	plas: 6, E.R.: 4, nucl: 2, cyto: 1
PtPHO7	Potri.010G164900	PHO	Chr10:16885666-16889577	2292	763	88.7	9.47	−0.246	11	9	10	plas: 10, E.R.: 2, nucl: 1
PtPHO8	Potri.010G165300	PHO	Chr10:16905446-16909093	2409	802	93.5	9.33	−0.192	12	10	10	plas: 10, chlo: 1, nucl: 1, E.R.: 1
PtPHO9	Potri.010G165800	PHO	Chr10:16935711-16945240	2388	795	92.8	9.29	−0.197	12	10	11	plas: 10, cyto: 1, E.R.: 1, pero: 1
PtPHO10	Potri.016G032900	PHO	Chr16:1872547-1876343	2325	774	90.5	9.48	−0.177	13	10	10	plas: 11, chlo: 1, E.R.: 1
PtPHO11	Potri.017G086800	PHO	Chr17:10433862-10439838	2379	792	90.6	9.26	−0.064	13	10	11	plas: 12, vacu: 1
PtPHO12	Potri.T146700	PHO	scaffold_467:326-6065	1890	629	72.8	9.32	−0.209	12	7	7	E.R.: 4, nucl: 3, chlo: 2, cyto: 2, plas: 2

**Figure 1:**
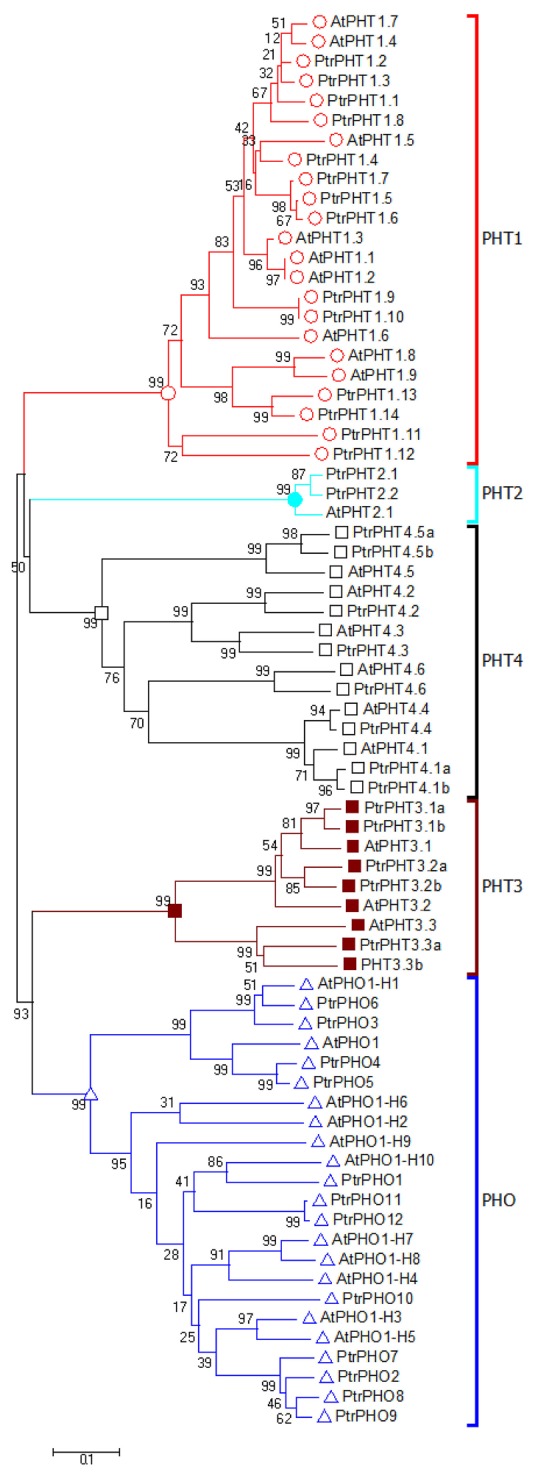
**Phylogenetic tree of ***P. trichocarpa*** and ***A. thaliana*** PHT proteins**. The phylogenetic tree was constructed using the neighbor-joining method with the 1000 bootstrap in MEGA 6 (http://www.megasoftware.net/).

The amino acid sequences of all of the *PHT* genes of *P. trichocarpa* were aligned with those of *A. thaliana*. The resulting phylogenetic tree contained five clusters with PHO, PHT1, PHT2, PHT3, and PHT4, respectively (Figure 1). Based on the phylogenetic analysis results, the *P. trichocarpa* PHT genes were named after the *A. thaliana PHT* genes (Figure 1, Table 1).

### Chromosomal location and structural analyses of *PtPHT* genes

**Figure 2:**
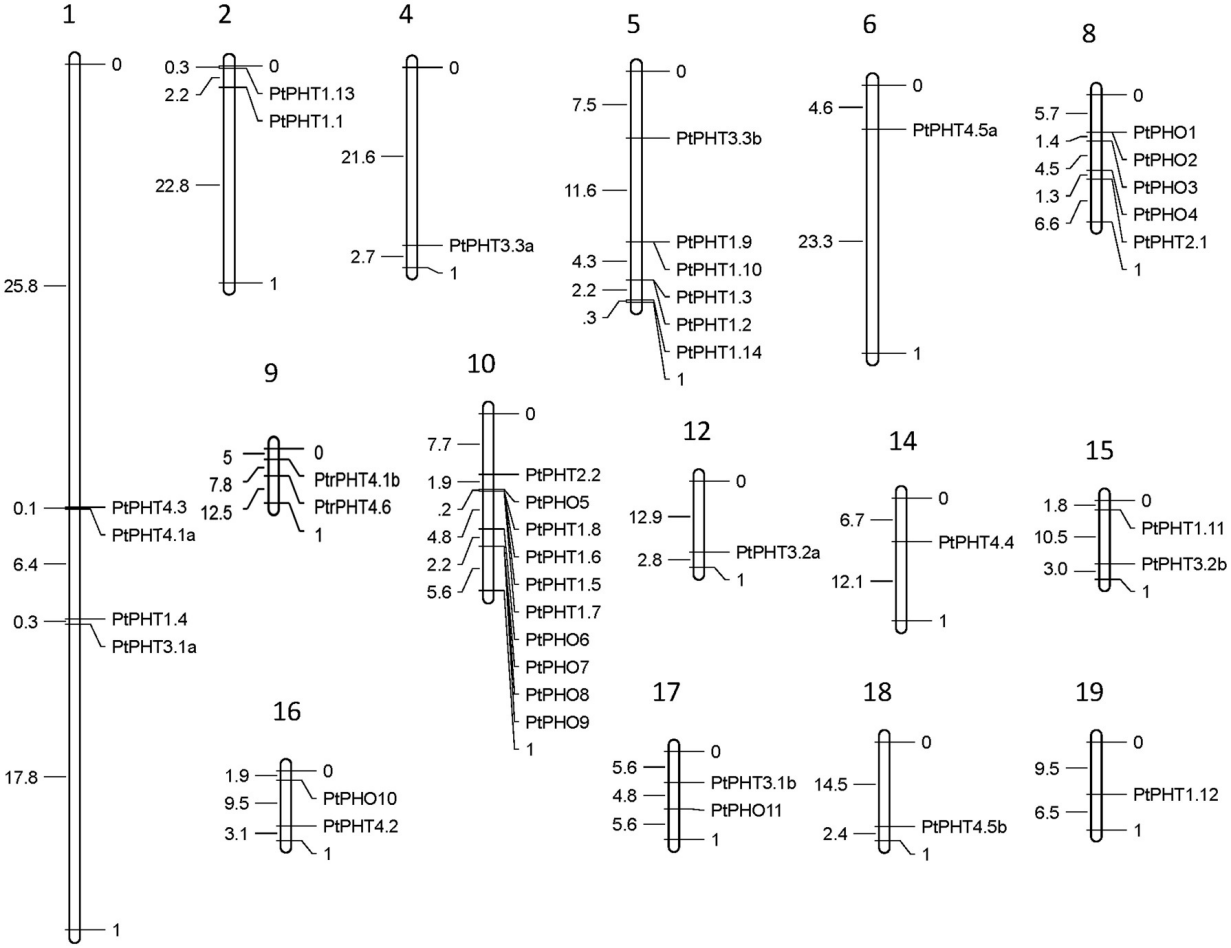
**Chromosomal locations of ***Populus PHT*** family members**. The chromosomal locations of the *PHT* genes were mapped with MapDraw program (Liu and Meng, 2003).

The *PtPHT* genes were marked on a physical map of *Populus* linkage groups based on the location information for *PHTs* in Phytozome 11.0. The *Populus PtPHT* genes showed a heterogeneous distribution pattern among the chromosomes (Figure 2). A total of 41 of the 42 *PtPHT* genes were localized to 15 of the 19 linkage groups of *Populus* (Figure 2). Only *PtPHO12* was localized to unattributed scaffold fragments.

The lengths of the 42 *PtPHT* genes ranged from 945 to 2409 bp, and they contained 1 to 16 exons (Table 1). These genes were distributed across 15 of the 19 poplar chromosomes excluding chromosomes 3, 7, 11, and 13 (Table 1, Figure 2). In addition, the lengths of the 12 *PtPHO* genes ranged from 1890 to 2409 bp, and they contained 11 to 14 exons (Table 1). Most of these genes were localized to chromosomes 8 and 10 (Table 1). Further, the lengths of the 14 *PtPHT1* genes ranged from 1311 to 1620 bp, and they contained 1 to 3 exons. Most of these genes were located on chromosomes 5 and 10 (Table 1). Two members of the PHT2 family, *PtPHT2.1* and *PtPHT2.2*, had lengths of 963 and 1773 bp and contained 2 and 3 exons, respectively. The PHT3 family included six *PtPHT* genes in poplar, with lengths ranging from 945 to 1125 bp and containing 6 exons. Further, the PHT4 family included nine *PtPHT* genes, with lengths ranging from 864 to 1662 bp and containing 1 to 11 exons.

**Figure 3:**
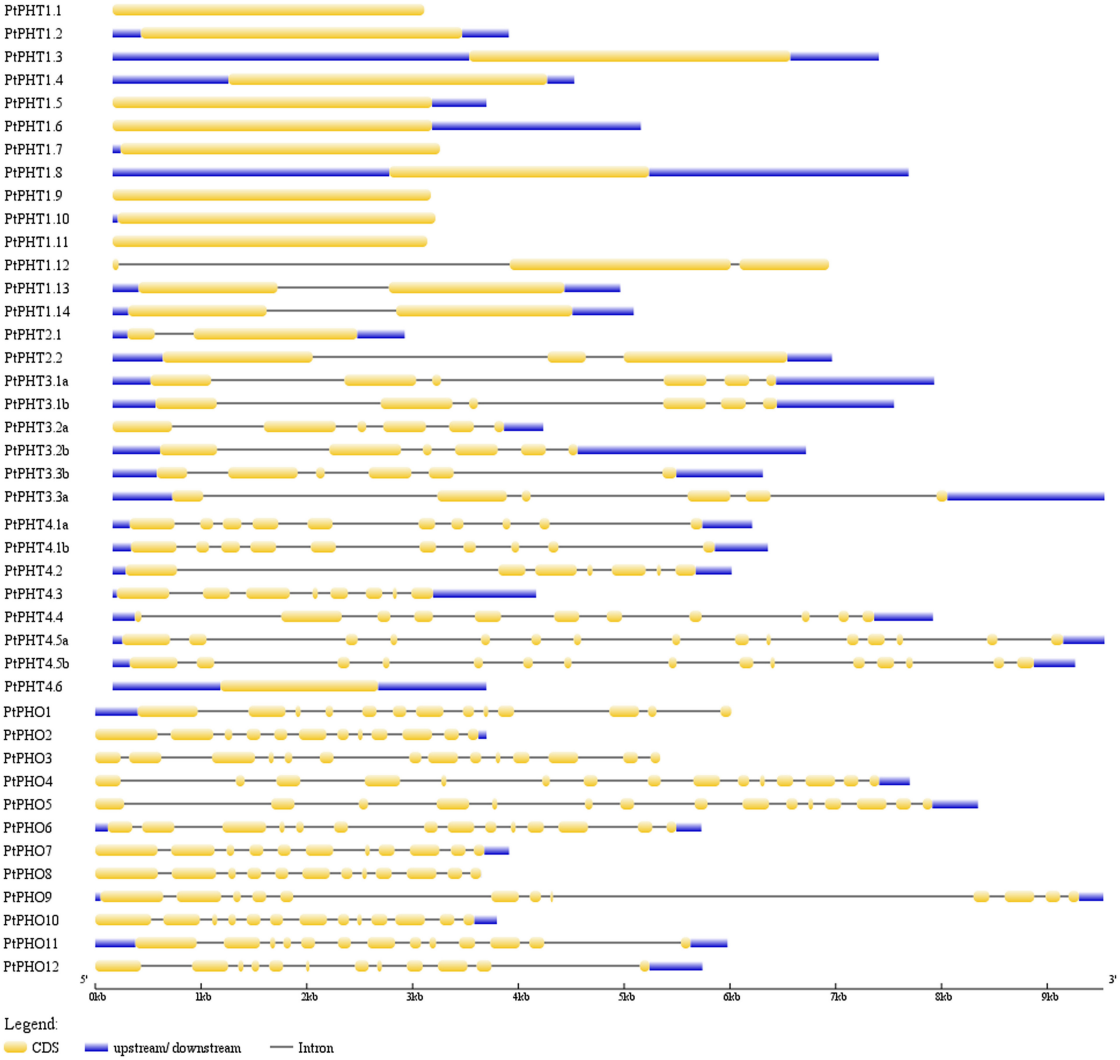
**Exon/intron structures of PHT genes in *Populus***. The exon/intron organization of the individual *PHT* gene was drawn using Gene Structure Display Server (GSDS) program (Hu et al., 2015).

We further analyzed the exon/intron structures of the 42 *PtPHT* genes. The genes belonging to the same PHT family, with the exception of the PHT2 family, were well conserved in terms of their exon/intron structures, with similar exon lengths (Figure 3). Unexpectedly, compared with other members of the PHT4 family, which contained 7–16 exons, *PtPHT4.6* possessed only one long exon, and its coding sequence was shorter. These substantial differences in gene structure are due to differences in the numbers of exons and introns among the various genes.

### *cis*-regulatory elements of *PtPHT* genes in poplar

The 2 kb upstream regions of the 42 *PtPHT* genes were analyzed using PLACE signal scan program. A total of 234 putative *cis* elements were detected, many of which are involved in development, stress, or hormone activity (Table S2). Twenty-six of the detected *cis* elements were found in all of the *PtPHT* genes involved in energy, light, hormone, defense, carbon metabolism, and tissue-specific expression. For example, GATABOX, GT1CONSENSUS, and IBOXCORE are light-responsive *cis* elements, and CACTFTPPCAL is carbon metabolism related *cis* element (Gowik et al., 2004).

Some Pi-response and other stress-related *cis* regulatory elements including ABRE elements, helix–loop–helix elements, PHO-like, PHR1, TATA-box-like, and WRKY1 were detected in the upstream regions of *PtPHT* genes. AACACOREOSGLUB1, a WRKY1 element, and ACGTABREMOTIFA2OSEM, an ABRE element, were present in 22 and 7 *PtPHT* genes, respectively. In addition, A PHO-like element (AACACOREOSGLUB1) and a TATA-box-like element (TATABOX2) was detected in 22 and 34*PtPHT* genes, respectively; A PHR1 element (P1SB) was observed in 20 *PtPHT* genes; and a helix–loop–helix element, (CATATGGMSAUR) was present in 11 *PtPHT* genes. Furthermore, 17 *PtPHT* genes had typical *cis* elements that were present especially in the respective genes, and the total number of these *cis* elements was 32.

### Transcription patterns of *PtPHT* genes in various tissues

**Figure 4:**
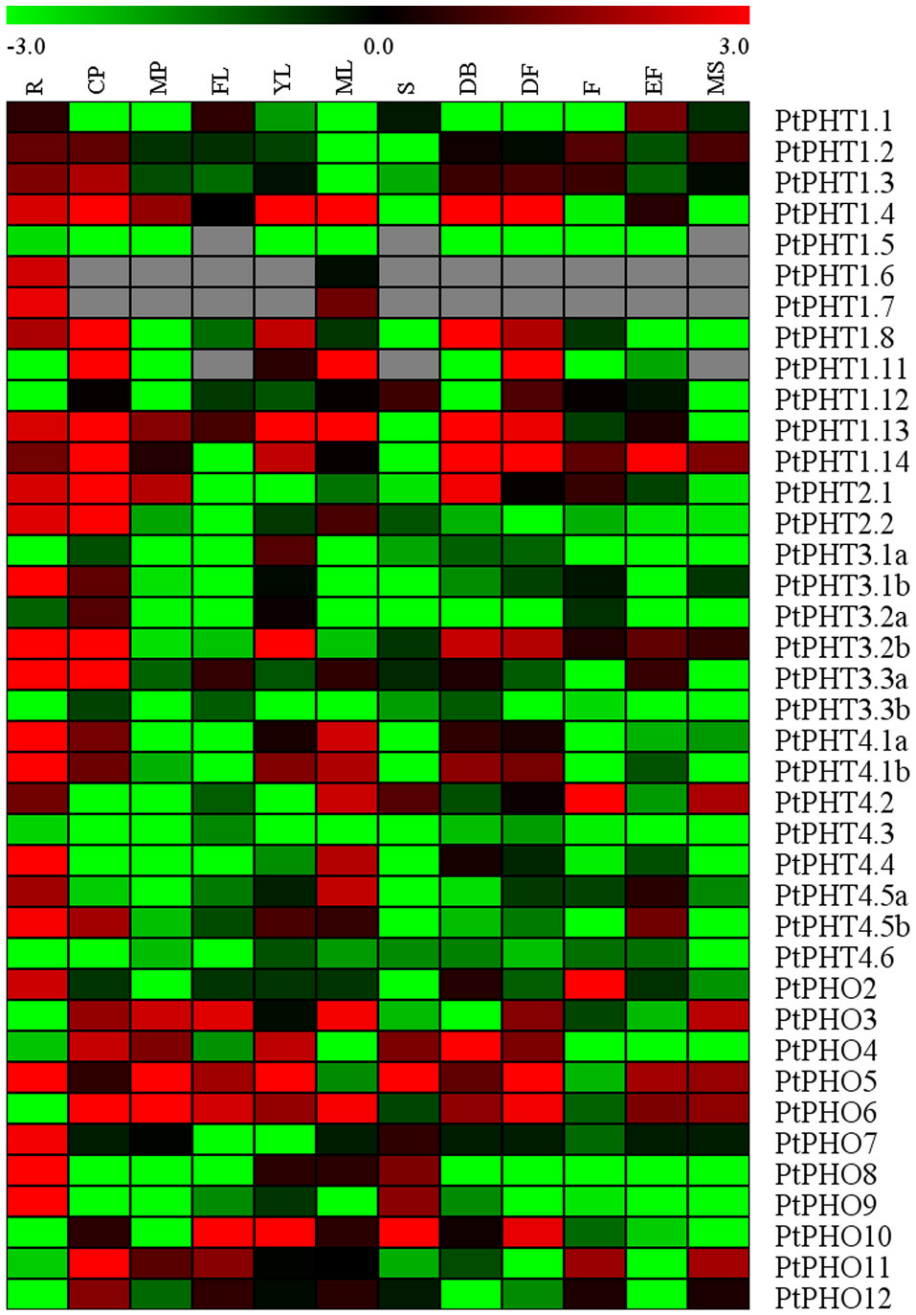
**The expression profiles of ***PHT*** genes in different tissues of ***Populus tremula*****. Heatmap shows PHT gene expression across 12 various tissues including roots (R), dormant cambium-phloem (CP), mature petiole (MP), freshly expanded leaves (FL), expanding young leaves (YL), mature leaves (ML), suckers (S), dormant buds (DB), dormant flowers (DF), expanded flowers (F), expanding flowers (EF), and mature seeds (MS). The data were obtained from PopGenIE (http://popgenie.org/) (Sjodin et al., 2009).

The specificities of tissues or organs may allow for the detailed elucidation of their functions. We measured the transcript levels of all of the *PtPHT* genes in 12 various tissues of *Populus tremula* using a public database, PopGenIE (http://popgenie.org/) (Figure 4). These tissues included roots, dormant cambium-phloem, mature petiole, freshly expanded leaves, expanding young leaves, mature leaves, suckers, dormant buds, dormant flowers, expanded flowers, expanding flowers, and mature seeds. Twenty-five *PtPHT* genes were highly expressed in the roots of *P. tremula*, suggesting that most *PtPHT* genes might be involved in Pi uptake. Six *PtPHT* genes, including *PtPHO3, PtPHO4, PtPHO5, PtPHO6, PtPHT1.4*, and *PtPHT1.14*, were highly expressed in more than six of the twelve investigated tissues. For example, *PtPHO6* was highly expressed in nine tissues, including cambium, petiole, expanded freshly leaves, expanding young leaves, mature leaves, dormant buds, dormant flowers, expanding flowers, and mature seeds. In contrast, the expression levels of several *PtPHT* genes, including *PtPHT4.3* and *PtPHT3.3b*, were very low in all of the investigated tissues. In particular, *PtPHT1.9* and *PtPHT1.10* were not expressed in any of the 12 tissues examined; this finding is not shown in Figure 4. Further, some *PtPHT* genes were only highly expressed in a few specific tissues. For example, *PtPHT1.6* was exclusively expressed in the roots of *P. tremula*, while *PtPHT1.5* and *PtPHO9* were only highly expressed in the roots and suckers of *P. tremula*.

**Figure 5:**
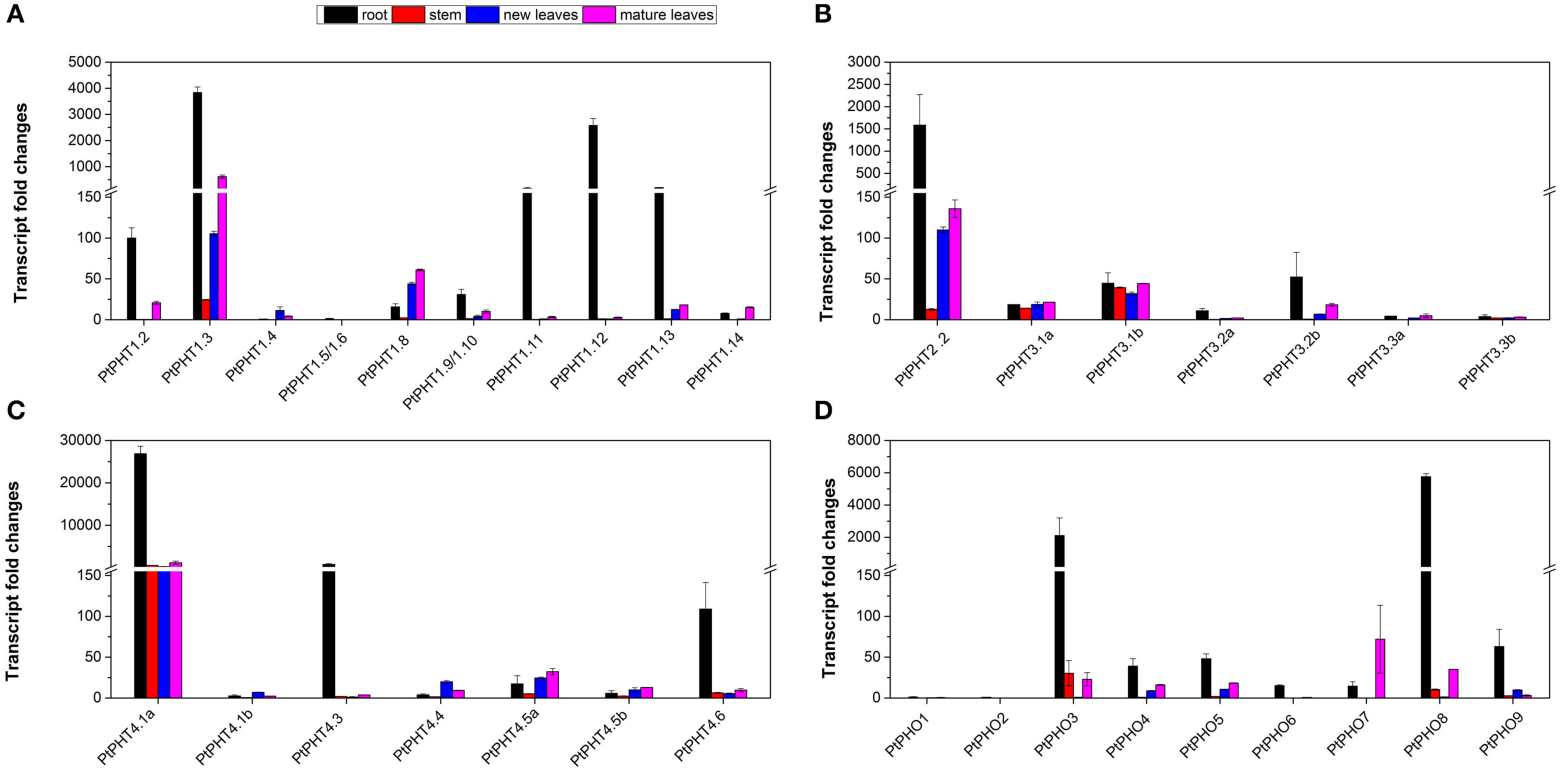
**The expression of ***PtPHT*** genes (A: PHT1; B: PHT2 and PHT3; C: PHT4; D: PHO) in different tissues of ***Populus simonii*****. Total RNA was isolated using a plant RNA extraction kit (R6827, Omega Bio-Tek, GA, USA) according to the manufacturer's instructions. The relative expression of PHT1.2 was set as a standard (100). The RNA was used for qRT-PCR with *PHT*-specific primers. The signal intensities were calibrated based on a constitutively expressed poplar actin gene (Brunner et al., 2004b). The data are presented as the mean ± SD of three separate measurements.

Real-time quantitative RT-PCR was also performed to analyze the transcript levels of all 42 *PtPHT* genes in the roots, stems, young leaves, and mature leaves of *P. simonii*. Transcripts of 35 *PtPHT* genes were detected in the examined tissues (Figure 5), but some of them were present at relatively low levels (such as *PtPHT1.5/1.6, PtPHO1*, and PtPHO*2*) (Figures 5A,D). Most of the *PtPHT* genes were confirmed to be expressed in the roots of poplar. Transcripts of 7 *PtPHT* genes (such as *PtPHT1.3, PtPHT2.2*, and *PtPHT4.1a*) were detected in all four tested tissues (Figures 5A–D). Most *PtPHT* genes were expressed in the roots of *P. simonii* at high levels. *PtPHT1.8* and *PtPHO7* exhibited increased transcript accumulation in the leaves compared to the roots (Figures 5A,D). In addition, *PtPHT3.1a*/*b* expression was similar in all four investigated tissues (Figure 5B).

### *PtPHT* transcript patterns in response to different phosphate levels

**Figure 6:**
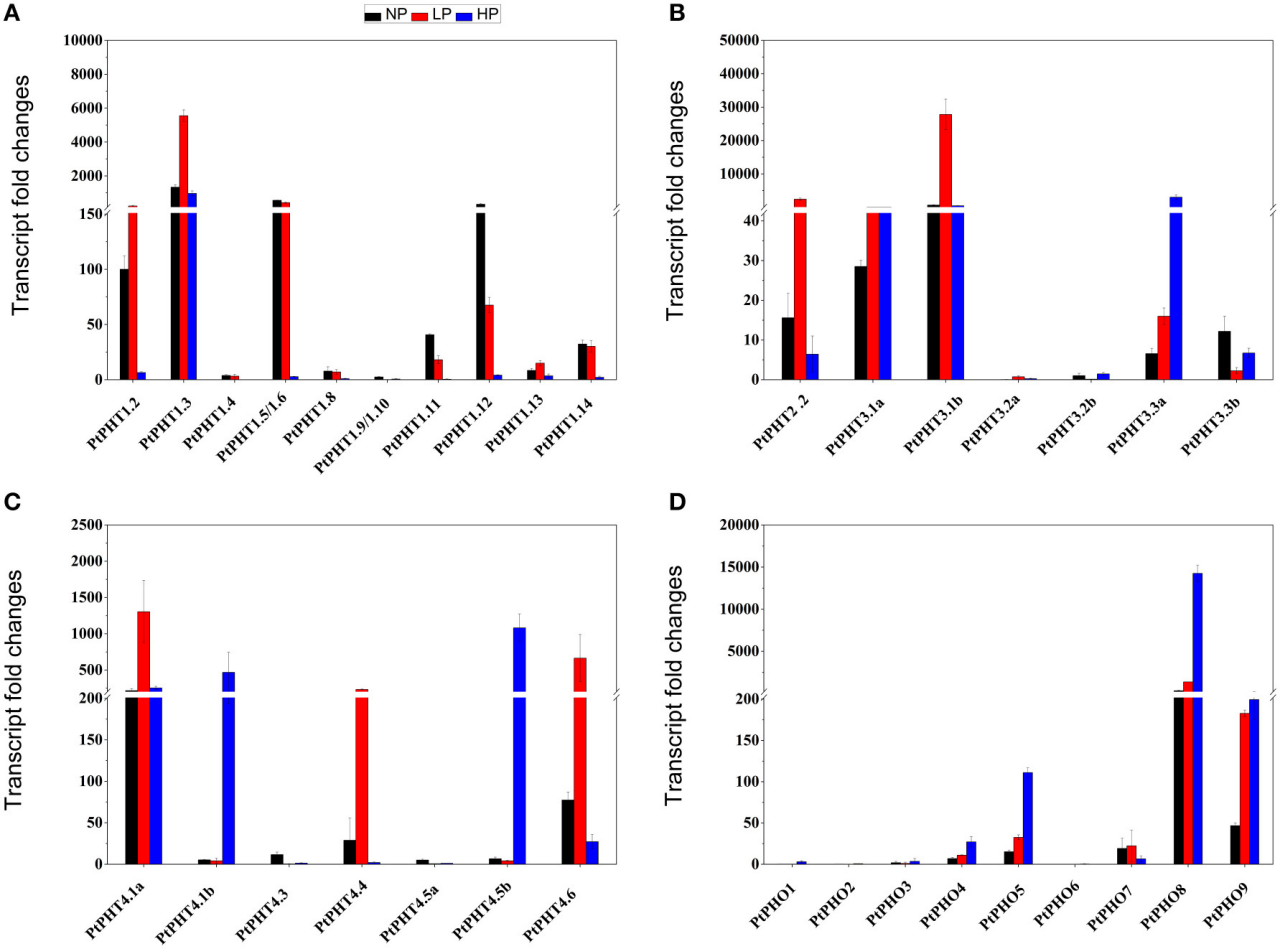
**The expression of ***PtPHT*** genes (A: PHT1; B: PHT2 and PHT3; C: PHT4; D: PHO) under different phosphate levels in roots of ***P. simonii*** seedlings**. The relative expression of PHT1.2 was set as a standard (100). Total RNA was isolated using a plant RNA extraction kit (R6827, Omega Bio-Tek, GA, USA) according to the manufacturer's instructions. The RNA was used for qRT-PCR with *PHT*-specific primers. The signal intensities were calibrated based on a constitutively expressed poplar actin gene (Brunner et al., 2004b). The data are presented as the mean ± SD of three separate measurements.

To better understand the functions of *PtPHT* genes in relation to phosphate uptake, the transcription patterns of *PtPHT* genes in the roots of *P. simonii* were examined at varying phosphate levels. There were 33 *PtPHT* genes in total expressed in roots (Figure 6), but the expression of a few *PtPHTs*, such as *PtPHT3.2a/b* and *PtPHO1/2/3*, was very low (Figures 6B,D). In the roots, the expression of *PtPHT1.12* was up-regulated and that of *PtPHT1.5* was only slightly altered under phosphate starvation conditions; in addition, the expression of *PtPHT1.2, PtPHT1.3, PtPHT2.2, PtPHT3.1b*, and *PtPHT4.1a* was markedly up-regulated under low-phosphate conditions, and that of *PtPHT3.3a, PtPHT4.1b, PtPHT4.5b, PtPHO8*, and *PtPHO9* was up-regulated under high-phosphate conditions (Figures 6A–D).

Most of the *PtPHT* genes were expressed at high levels in the roots of *P. simonii* in the presence of low phosphate levels, indicating that they are high-affinity genes. The accumulation of transcripts of many *PtPHT* genes was reduced when the level of H_2_${\text{PO}}_{3}^{-}$ was increased in the roots of *P. simonii*, with the exception of *PtPHO8* and *PtPHO9*.

### Changes in *PtPHT* transcription in response to drought stress

**Figure 7:**
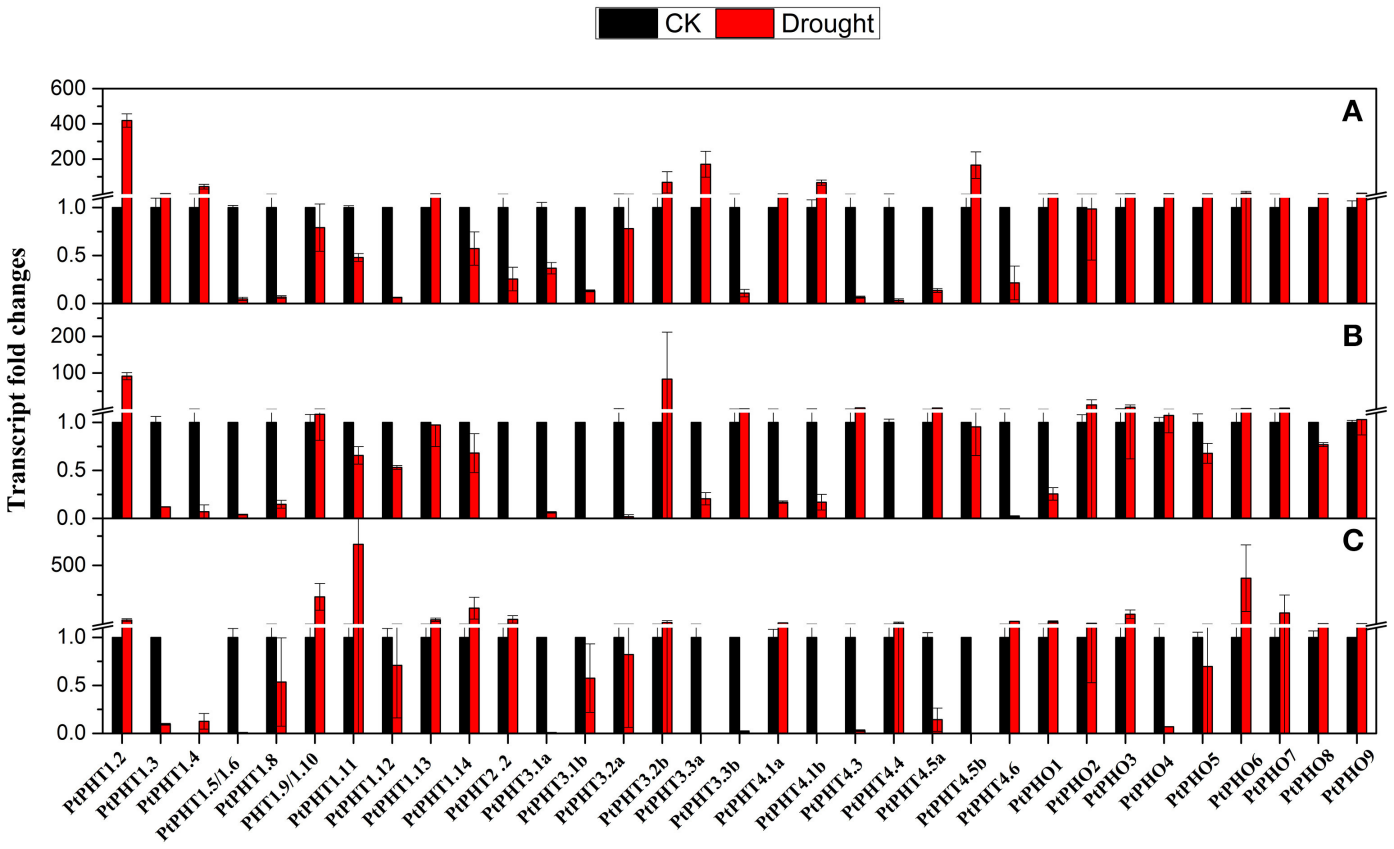
**The expression of ***PHT*** genes affected by drought at different phosphate levels (A: No P; B: low P; C: high P) in roots of ***P. simonii*** seedlings**. The control is defined as 1 in the figures. Total RNA was isolated using a plant RNA extraction kit (R6827, Omega Bio-Tek, GA, USA) according to the manufacturer's instructions. The RNA was used for qRT-PCR with *PHT*-specific primers. The signal intensities were calibrated based on a constitutively expressed poplar actin gene (Brunner et al., 2004b). The data are presented as the mean ± SD of three separate measurements.

To understand the transcriptional changes of *PtPHT* genes induced by drought, *P. simonii* plants supplied with different phosphate levels were subjected to drought stress for 2 weeks. *PtPHT1.2* and *PtPHO9* expression was found to be increased under drought conditions, irrespective of the phosphate level. In particular, *PtPHT1.2* expression was significantly induced by approximately 90-fold. However, changes in the transcription of some *PtPHT* genes under drought stress were largely dependent on the phosphate level. For example, after 14 days of drought stress, the expression of *PtPHT3.3b, PtPHT4.3*, and *PtPHT4.5a* was significantly down-regulated, while that of *PtPHT1.9, PtPHO4*, and *PtPHO9* was only slightly increased when no phosphate was supplied (Figure 7). However, the expression of these genes was up-regulated under drought stress in the presence of a low level of phosphate. Further, in the presence of a high level of phosphate, the expression of *PtPHT1.9, PtPHT1.11, PtPHT1.3, PtPHT1.14, PtPHT2.2, PtPHT4.4, PtPHT4.6, PtPHO1*, and *PtPHO8* was increased under drought conditions. These data suggested that the changes in the transcription of some *PtPHT* genes under drought conditions were strongly influenced by the phosphate level.

## Discussion

Phosphorus is a mineral nutrient that is essential for plant growth and development. Pi was assimilated in roots via PHT transporters. In this study, we identified the whole PHT gene family in poplar and examined the expression patterns of these genes in different plant tissues and in response to differing Pi levels and drought. The PHT family, the *cis* elements of the PHT genes, and transcriptional changes in the PHT genes in response to different Pi levels and drought are discussed below.

### PHT gene family in *populus*

In the current study, we conducted comprehensive analysis of PHT genes in poplar. A total of 42 *PtPHT* genes were retrieved from *Populus* genome (Phytozome 11.0, *P. trichocarpa* v3.0) with improved annotation, and they were classified into five clusters: PHT1, PHT2, PHT3, PHT4, and PHO. In a subsequent study, Loth-Pereda et al. (2011) reported the identification of 12 PHT1 family members in an earlier version of the *Populus* genome (v1), and we expanded upon their findings with the identification of 14 PHT1 family members in the latest version of *Populus* genome (v3). Notably, the PHT1 family is larger in poplar (14 genes) than in *Arabidopsis* (9 genes), barley (8 genes), rice (13 genes), and tomato (8 genes) (Muchhal et al., 1996; Rae et al., 2003; Liu et al., 2011; Chen et al., 2014).

The PHT2, PHT3, PHT4, and PHO families contained more members of *PtPHT* genes in poplar than that in *Arabidopsis*. One PHT2 family gene has been identified in *Arabidopsis* (Daram et al., 1999), while two (*PtPHT2.1* and *PtPHT2.2*) were detected in poplar. These two genes are located on different chromosomes (Chr8 and Chr10), and the lengths of their encoded proteins greatly differ which is constituent with the previous report that *AtPHT2.1* cDNA encodes a 61-kD protein that is structurally similar but different from the PHT1 family proteins in *Arabidopsis* (Daram et al., 1999). However, phylogenetic analysis showed that *PtPHT2.1* and *PtPHT2.2* share 100% similarity (Table S3). At least three *AtPHT3* genes have been identified in *Arabidopsis* to date (Rausch and Bucher, 2002), while we found six PHT3 family genes in poplar. Further, six PHT4 family genes have been characterized in *Arabidopsis* (Guo et al., 2008), while 8 *PtPHT4* genes were detected in the present study. Ten genes with homology to *AtPHO1* are present in the *Arabidopsis* genome (Hamburger et al., 2002), and in this study, 12 PHO family genes were detected with similarities to the PHO1 protein in *Arabidopsis*.

The presence of a large number of genes within a family reflects the successive expansion and rearrangement of the genome by the extensive duplication and diversification that frequently occur over the course of evolution (Wang et al., 2007). The *P. trichocarpa* genome is duplicated and contains more protein-coding genes than the *Arabidopsis* genome; on average, there are 1.4 to 1.6 putative *Populus* genes for every *Arabidopsis* gene (Tuskan et al., 2006). Whole-genome duplication increases gene gains and losses, which greatly affects the amplification of members of gene families in the genome (Guo, 2013). The expanded genes in the PHT family, including PHT1, PHT2, PHT3, PHT4, and PHO, in poplar compared with *Arabidopsis* might be the result of gene duplication. Gene duplication has been reported in *P. trichocarpa* in many studies, including studies of the glutamine synthetase family, nitrate transporter gene family, and CCCH zinc finger family (Castro-Rodríguez et al., 2011; Chai et al., 2012; Bai et al., 2013). In the present study gene expansion and loss might also occur in the PHT family of *P. trichocarpa* similar to that of *Arabidopsis*. For example, two closely related orthologs of *Arabidopsis, AtPHT4.1, AtPHT4.5*, and *AtPHO1*, were found in *Populus* suggesting that duplications might be a partial reason for the expansion of PHT gene family in *Populus*. Forty-three *PtPHT* genes were localized to 15 poplar chromosomes, and some *PHT* genes were found to be clustered together as closely related genes (Figure 2). The presence of these closely related clusters suggests that these genes might evolve from regional duplications (e.g., *PtPHO6, PtPHO7, PtPHO8*, and *PtPHO9*).

### The *cis* elements identified in poplar

*Cis* elements may control the efficiency of promoters and thus regulate the expression of the genes that they control by interacting with the corresponding *trans*-regulatory factors (Liu et al., 2011). Studies of *cis* elements might play a crucial role in dissecting the functions of genes. A previous study has described the *cis* elements of PHT1 family members in poplar (Loth-Pereda et al., 2011), but the *cis* elements of all PHT gene family members in poplar have not yet been identified. In this study, the upstream regions of all the *PtPHT*s contain *cis* elements affecting energy, light, hormone, defense, carbon metabolism, and tissue-specific expression. These findings suggest that the expression of these *PtPHT* genes might be regulated by many factors, such as light-, hormone-, and defense-related factors. For instance, GATABOX and GT1CONSENSUS are *cis* elements required for light regulation (Lam and Chua, 1989; Terzaghi and Cashmore, 1995), and they have also been identified in the upstream regions of all *OsPHT* genes in rice (Liu et al., 2011). Further, putative PHO-like (CGCGTGGG) and TATA-box-like (TATAAATA) *cis* regulatory elements have been identified in the promoter regions of *Arabidopsis* genes (Hammond et al., 2003), and they were observed in 22 and 34 of the 42 *PtPHT* promoters, respectively. Alternatively, because the core sequence of the *Arabidopsis* PHO-like element (CACGTG) is also similar to that of the ABRE element (ABREOSRAB21), which is present in 6 of the 42 *PtPHT* promoters, proteins in the bZIP class of transcription factors might bind to them (Schindler et al., 1992). The presence of these *cis* elements in poplar *PtPHT* genes and their likely roles in regulating gene expression suggest that *PtPHT* genes may be involved in various stress responses in poplar.

Some *cis* elements function under phosphate deficiency, for example, the P1BS element can be bound to PHR1 (a MYB transcription factor) to potentially regulate the phosphate starvation response in *Arabidopsis* (Rubio et al., 2001), PHR1 has been suggested as a central integrator to role in the transcriptional regulation of phosphate starvation responses (Bustos et al., 2010). In the current study, P1BS-like elements were found in 20 of the 42 *PtPHT* promoters examined suggesting that expression of these genes might be influenced by Pi starvation. Similarly, P1BS-like elements have also been detected in *OsPHT* genes in rice (Liu et al., 2011). These elements are found to be in the promoters of phosphate regulated genes, not only in *Arabidopsis* but also in other plant species such as barley and rice (Schünmann et al., 2004; Liu et al., 2011), which suggests that they may participate in a conserved signaling pathway for the phosphate starvation response in plants. The existence of P1BS-like elements in poplar, rice, and *Arabidopsis* suggests that they may serve a similar role in both woody species and herbs.

### Transcript profiles of *PHT* genes in various tissues

The number of Pi transporters present in roots may reflect the complexity and significance of the process of Pi transport. In the current study, most PHT family members were expressed in the roots of *P. tremula* and *P. simonii*. In particular, most PHT1 family members were highly expressed in the roots of poplar, consistent with a previous study conducted by Loth-Pereda et al. (2011) suggesting that they may function in Pi uptake. Notably, most *PHT1* genes in other plant species are also expressed in the roots. For example, eight of the nine *PHT1* family genes in *Arabidopsis* are expressed in the roots (Bucher et al., 2001; Mudge et al., 2002), in addition, 8 *PHT1* genes in tomato (Chen et al., 2014) and 10 of the 13 *PHT1* genes in rice are expressed in the roots (Paszkowski et al., 2002). These results indicate that *PHT1* family genes may play similar roles in Pi uptake in different species.

Homologous *PHT1, PHT2, PHT3, PHT4*, and *PHO* genes showed substantial differences in their regulation in different tissues. Nine PHT1 family genes were highly expressed in the roots of *P. tremula*, but the expression of the other five genes was very low, and two of these genes (*PtPHT1.9* and *PtPHT1.10*) were not expressed in any tissues examined. *PtPHT2.1* was highly expressed in the roots, cambium-phloem, mature petiole, and dormant buds, while *PtPHT2.2* was mainly expressed in the roots, cambium-phloem, and mature leaves. Six homologous *PtPHT3* genes exhibited substantial differences in their expression in the various tissues examined. Three of them (*PtPHT3.1b, PtPHT3.2b*, and *PtPHT3.3a*) were highly expressed in the roots of *P. tremula*, while the others (*PtPHT3.1a, PtPHT3.2a*, and *PtPHT3.3b*) were expressed at very low levels. Although most PHT4 family members, excluding *PtPHT4.3* and *PtPHT4.6*, were highly expressed in the roots and leaves, *PtPHT4.1* was also highly expressed in the cambium-phloem, dormant buds, and dormant flowers. Further, *PtPHO5* and *PtPHO6* were highly expressed in nine tissues examined, while *PtPHO7* and *PtPHO8* were exclusively highly expressed in only a few of the tissues. Expression of the *PtPHT* genes varied among the different tissues examined, suggesting that these genes may have different functions in Pi uptake, transport, and storage. These results suggest that the *PHT* genes may have different functions since they expressed in a wide range of tissues, which may be partly crucial for the growth of perennial species.

The differential expression of *PtPHT* genes in different tissues might be related to their subcellular locations (Table 1). For example, two PHT2 family members were expressed in the leaves of *P. simonii*, in agreement with a previous study demonstrating that the *AtPHT2.1* gene, which is located in the chloroplast, is predominantly expressed in green tissues in *Arabidopsis* (Liu et al., 2011). In addition, the expression patterns of *PtPHT4* genes in both roots and leaves, and the phosphate transport activities as well as their subcellular locations, indicate that PHT4 proteins play roles in transferring Pi between the cytosol and chloroplasts, heterotrophic plastids, and the Golgi apparatus (Guo et al., 2008). For example, *AtPHT4.6* transports Pi out of the Golgi lumenal space to recycle Pi released from glycosylation processes in *Arabidopsis* (Cubero et al., 2009). In addition, the PHO1 protein has been reported to be localized to stellar cells of the roots as well as the lower part of the hypocotyl in *Arabidopsis* (Ribot et al., 2008). In the present study, PHO family members were found to be highly expressed in many of the tissues examined, suggesting that they have diverse functions in loading Pi to different parts of plants.

### Transcript profiles of *PHT* genes in response to different phosphate levels and drought in *populus*

The transcription of *PtPHT* genes differed at different Pi levels. Upon Pi starvation, *PtPHT1.9, PtPHT1.11, PtPHT1.12, PtPHT3.3b, PtPHT4.3*, and *PtPHT4.5a* expression was up-regulated in *P. simonii* (Figure 5). The up-regulation of some *PtPHTs*, such as *PtPHT9* and *PtPHT11*, under Pi starvation is consistent with the findings of a previous study of *P. trichocarpa* conducted by Loth-Pereda et al. (2011). Most PHT1 family genes expressed in roots show up-regulation in phosphate deprived *Arabidopsis* plants; in particular, *PtPHT9* and *PtPHT11* in poplar are highly homologous to *AtPHT8* and *AtPHT9*, which have been shown to be strongly up-regulated during Pi starvation (Mudge et al., 2002). In rice, high-affinity Km values of *OsPHT9* and *OsPHT10* for Pi transport have been reported, as well as specific induction of their expression by Pi starvation (Wang et al., 2014). Therefore, some of these PHT transporters likely play roles in Pi scavenging under conditions of low soil Pi availability. *PtPHT1.2, PtPHT1.3, PtPHT2.2, PtPHT3.1b*, and *PtPHT4.1*a expression was markedly up-regulated under low-Pi conditions, suggesting that their expression might be induced under these conditions. The transcript levels of a few PHT genes in *Arabidopsis*, such as *AtPHO1, AtPHO;H1*, and *AtPHO;H10*, were increased by Pi deficiency (Ribot et al., 2008); however, the expression of most PHO family members was not induced by Pi deficiency in poplar, suggesting that *PHT* genes might have different functions in woody species and herbs. *PtPHT3.3a, PtPHT4.1b, PtPHT4.5b, PtPHO8*, and *PtPHO9* expression was up-regulated under high-phosphate conditions, indicating that these proteins might be low-affinity transporters. In addition, some PHT4 family members might be low-affinity transporters, consistent with the relatively high Pi levels that have been reported in the cytosol and other subcellular compartments (Mimura, 1999).

The expression of *PHT* genes varied at different Pi levels, suggesting that PHT family members are likely to encode both high- and low-affinity transporters. For example, *OsPHT2* shows low-affinity characteristics on the mM scale in oocytes (Ai et al., 2009). By contrast, a Km value of 19 μM has been reported for *HvPHT1* (barley high-affinity transporter, Preuss et al., 2010). *AtPHT1.1* has been demonstrated to be a high-affinity transporter in a gene over-expression study (Km value of 3.1 μM, Mitsukawa et al., 1997), in addition to *HvPHT1.1* (9.06 μM) and *HvPHT1;6* (385 μM) in barley (Rae et al., 2003). The affinities of *PHT* genes in poplar require further study, including assessment of the heterologous transformation of *Xenopus* oocytes and gene over-expression studies using plant cell cultures.

The expression levels of some *PtPHT* genes, such as *PtPHT1.2* and *PtPHO9*, were up-regulated under drought conditions, irrespective of the Pi level, suggesting that their expression was not affected by the Pi level under these conditions. However, the changes in expression of the other *PtPHT* genes, including *PtPHT3.3b, PtPHT4.3*, and *PtPHT4.5a*, under drought stress were largely dependent on the phosphate level (Figure 6). Especially, the upregulation of *PtPHT* genes induced by a low level of Pi may contribute to drought tolerance of poplar plants. These results suggest that under drought conditions, Pi uptake in plants likely changes in association with alterations in *PHT* gene expression. This altered uptake in the presence of different levels of phosphate might be related to the drought resistance of plants.

## Author contributions

CZ and ZZ conceived and designed the experiments. SM and CZ performed the experiments. CZ, ML, and SM analyzed the data. CZ, ML, and ZZ wrote the paper.

### Conflict of interest statement

The authors declare that the research was conducted in the absence of any commercial or financial relationships that could be construed as a potential conflict of interest.

## Supplementary material

Table S1 **The primers used for quantitative real time PCR**.

Table S2 **Cis elements of PHT genes in *Populus***.

Table S3 **The sequence similarity of PHT genes in *Populus***.
